# Inhibition of Hemorragic Snake Venom Components: Old and New Approaches

**DOI:** 10.3390/toxins2040417

**Published:** 2010-03-25

**Authors:** Isabella Panfoli, Daniela Calzia, Silvia Ravera, Alessandro Morelli

**Affiliations:** Department of Biology, University of Genova, Viale Benedetto XV 3, 16132 Genova, Italy; Email: dcalzia@gmail.com (D.C.); silvia.ravera@gmail.com (S.R.); morellia@unige.it (A.M.)

**Keywords:** antivenins, direct electric current, etnomedicine, metalloprotease, phospholipase A2, phosphodiesterase, snake venom

## Abstract

Snake venoms are complex toxin mixtures. Viperidae and Crotalidae venoms, which are hemotoxic, are responsible for most of the envenomations around the world. Administration of antivenins aimed at the neutralization of toxins in humans is prone to potential risks. Neutralization of snake venom toxins has been achieved through different approaches: plant extracts have been utilized in etnomedicine. Direct electric current from low voltage showed neutralizing properties against venom phospholipase A2 and metalloproteases. This mini-review summarizes new achievements in venom key component inhibition. A deeper knowledge of alternative ways to inhibit venom toxins may provide supplemental treatments to serum therapy.

## 1. Geographic Distribution of Venomous Snake Families

Snake venoms are complex mixtures of biologically active proteins, peptides, metal ions and organic compounds, which have evolved to favor the survival of the snake in its particular environment [1]. There are more than 600 known species of venomous snakes—about a quarter of all snake species—classified into several families: Elapidae, Viperidae, Crotalidae, Hydrophidae, Atractaspididae and Colubridae [2]. Their venoms are classified as hemotoxic if they primarily affect the cardiovascular system, or neurotoxic if they affect the central nervous system (CNS) and muscular system [3]. 

In Central and South America, the United States (USA), Australia, India and Africa, about 2.5 million people are bitten by snakes annually and more than 100,000 fatally [4,5,6,7,8]. Most morbidity and mortality occurs in rural areas in the tropics [9], with farmers, hunter gatherers, herdsmen and children being the major groups at risk. In the USA, every state but Maine, Alaska and Hawaii is home to at least one of 20 domestic poisonous snake species [10,11,12]. In the USA, 99 percent of snakebites result from envenomation with snakes of the family Viperidae or family Crotalidae (which includes rattlesnakes, copperheads and water moccasins) [11]. Snakes of the families Viperidae and Elapidae are also responsible for the high incidence of snake bites in West Africa, the Indian subcontinent, South-East Asia, New Guinea and Latin America [5,10]. Due to their widespread distribution and relatively potent venom, the fatalities caused by rattlesnakes are the most diffused and best documented. Pit vipers of the genus Bothrops are responsible for the majority of fatalities from snakebites in Latin America [11]. The Puff Adder, a common name of snake species of the genus *Bitis*, is considered Africa’s most dangerous snake as it is responsible for more fatalities than any other snake in Africa. In Europe, envenomation is mostly due to Viperidae [13]. The incidence of European venomous *Vipera* spp bites is however difficult to assess. Mortality is low, but small children and elderly people may face life-threatening situations. Exotic snakes, including venomous species, are becoming increasingly popular pets in Western countries. Some of them are kept illegally. Exotic snake-handlers, including venomous species, and their physicians face a major challenge in Western countries [14]. Table 1 summarizes the geographic distribution of the most represented families of hemorrhagic venomous snakes.

**Table 1:** Geographic distribution of hemorrhagic venomous snakes.

Families of hemorrhagic venom snakes	Geographic distribution
Elapidae	West Africa, South-East Asia, America, Australia and New Guinea.
Colubridae	Worldwide except Antarctica, extremely high latitudes of Eurasia and North America and central and western Australia
Crotalidae	Asia and America
Viperidae	Europe, Africa, South-East Asia and USA

## 2. Key Enzymes in Hemotoxic Venom Action

Venoms from Viperidae, Crotalidae and some Australian snakes affect the hemostatic mechanism or disrupt the endothelium [13,14]. Their venom contains serine-proteases, hemorrhagins (fibrinogenases possessing high anti-thrombotic activity), fibrinolytic activators , metalloproteases and group II PLA_2_ isoenzymes, as well as non-enzymatic proteins (C-type lectins, CRISP and disintegrins) that activate or inhibit coagulant factors or platelets, or disrupt the endothelium [15]. To date, thanks to the recent developments in proteomic techniques and mass spectrometry for the identification of proteins, the snake venom proteome and the pharmacological properties of its components are being explored [16]. Many snake venom toxins affect platelet function by inducing or inhibiting platelet aggregation [17]. Approximately 100 snake venom toxins have been identified as ‘thrombin-like’ enzymes activating the blood coagulation factor [18].

Neurotoxins are classified according to their site of action (*i.e.*, pre- or post-synaptic). Post-synaptic neurotoxins are antagonists of the nicotinic receptor on the skeletal muscle leading to paralysis and eventually death. They have only been identified in venoms from the families Elapidae and Hydrophiidae [18].

Phospholipase A_2_ (posphatidate 2-acylhydrolases, PLA_2, _EC 3.1.1.4). PLA_2_ are probably the most thoroughly investigated toxins both in hemotoxic and presynaptic neurotoxic snake venoms [19,20]. PLA_2_ has also been classified as a presynaptic neurotoxin, identified in the venoms of *Crotalidae*, *Elapidae*, *Hydrophiidae* and *Viperidae* snakes [19]. PLA_2_ are ubiquitous intra- and extra-cellular enzymes hydrolyzing glycerophospholipids at the *sn*-2 position of the glycerol backbone releasing lysophospholipids and fatty acids [20], in turn arachidonate metabolites control inflammation and pain [21]. PLA_2_ are responsible for the local inflammation following *viperid* snakebite envenomation. Venoms are rich sources of a large number of PLA_2_ isozymes [22], which can have pharmacological effects *in vivo* [23]. While mammalian PLA_2_ are generally nontoxic, snake venom enzymes or their complexes are the active component of both hemotoxic and presynaptic neurotoxic venoms of rattlesnakes and Australian elapid snakes [22,24], exhibiting a variety of pharmacological effects, through mechanisms that can also be independent of its enzymatic activity [3,23].

For hemotoxic venoms, conspicuous toxic consequence of snake envenoming is hemorrhage production, which can become systemic and potentially lethal. Hemorrhages are principally caused by metalloproteases (also called hemorrhagins), enzymes degrading proteins of extracellular matrix and components of the hemostatic system, that can also have cytotoxic effect on endothelial cells [25,26]. The majority of metalloproteases belong to the family of zinc endopeptidases grouped together as a superfamily known as zinc-dependent Snake Venom Metallo Proteinases (SVMP, also called metzincins or hemorrhagins, EC 3.4.24.-). The metzincins are subdivided into four multigene families: seralysins, astacins, ADAMs/adamalysins, and MMPs. On the basis of sequence similarity they share a highly conserved motif containing three histidines [27] that bind to zinc at the catalytic site and a conserved methionine that sits beneath the active site [28]. Examples are: adamalysin II (EC 3.4.24.46), atrolysin C/D (EC 3.4.24.42), trimerelysin I (EC 3.4.24.52) and II (EC 3.4.24.53) [29]. All metalloproteases contain approximately 1 mole of zinc per mole of toxin [27]. When zinc is removed from hemorrhagic toxins, for example with a chelator, proteolytic and hemorrhagic activities are simultaneously abolished due to structural alterations [30,31].

## 3. New and Old Approaches for Inhibition of Hemorrhagic Venoms

Envenomations due to snake bites are commonly treated by parenteral administration of horse or sheep-derived polyclonal antivenoms aimed at the neutralization of toxins. Although there is no universal grading system for snakebites, a I through IV grading scale has been developed for clinical use, as a guide to antivenin administration. First-aid measures for snakebite include avoiding excessive activity, immobilizing the bitten extremity, and quickly transporting the victim to the nearest hospital. Venomous snakes, even dangerous ones like the Eastern diamondback, do not always release venom when they bite. US medical professionals may not agree on every aspect of what to do for snakebite first aid, but they agree on what not to do: no cooling, tourniquets, incisions and no electric shock on the bite, however the protocols for assistance of the victims of envenomation are money and time consuming. 

Developing effective and cheap antivenins (sometimes called "antivenoms"), designing control assays, and recruiting the resources needed to validate them is an economic and ethic problem. Equine-derived antivenin is considered the standard of care; however, snakebite victims who are sensitive to horse proteins must be carefully managed. They could in fact develop an adverse reaction or even an anaphylactic shock [12]. A sheep antibody preparation (CroFab) is now licensed for use in the United States [32] and is a promising new treatment. CroFab is sheep-derived antigen binding fragment ovine, which is much less allergenic, being digested to reduce the risk of allergic reactions. The venom of the snake *Bothrops asper* has been the subject of a number of experimental studies addressing its neutralization by antibodies. Recently, a process for raising antibodies against *Bothrops* sp venom in chicken egg yolks from hens immunized with Brazilian standard bothropic antigen preparation was developed in Brazil. The technique yielded antibodies capable of neutralizing lethal toxic activity of the pool of *Bothrops* venoms from five species [33]. 

In the case of neurotoxic venoms, these cause little local tissue reaction, and the onset of the of clinical signs from neurotoxins (a curare like-syndrome) may be delayed for as much as 10 hours. Therefore, the treatment is exclusively done by administration of specific antivenins. Antivenin development may not be affordable in many countries [8] and there are few lucrative markets for producers. This lack of competitive commerce reduces the incentives for progresses in the production of new antivenins. Moreover, the phenomenon of intraspecific variation in snake venom composition must be kept in mind. Because not all snakebites, including those from the same species, are equally dangerous, doctors sometimes face a dilemma over whether or not to administer antivenin [32]. Despite the widespread success of antivenin therapy, it is still important to search for different venom inhibitors, either synthetic or natural, that could complement or substitute for the action of antivenins. 

### 3.1. Natural Venom Inhibitors

Natural inhibitors of snake venoms play a significant role in the ability to neutralize the degradation effects induced by venom toxins. It has been known for many years that animal sera and some plant extracts are competent in neutralizing snake venoms. Several plants have been utilized in folk medicine as antiophidians. However, only a few species have been scientifically investigated and still less have had their active components isolated and characterized both structurally and functionally. Protective activity of many of these plants against the lethal action of snake venoms has been confirmed by biological assays. Compounds present in all of them belong to chemical classes capable of interacting with macromolecular targets (enzymes or receptors). Biotechnological application of these inhibitors, may represent helpful alternative or supplemental treatments to serum therapy [34]. Several substances have been evaluated regarding their effects against snake venoms and isolated venom toxins, including plant extracts and compounds from marine animals, mammals and snake serum plasma, in addition to many synthetic molecules [35].

Several Brazilian plants have been utilized in folk medicine as active agents against snake venoms. The aqueous extract of *Pentaclethra macroloba* (EPema) exhibited full inhibition of hemorrhagic and nucleolytic activities induced by several snake venoms. *In vivo* tests showed that EPema is able to inhibit a *Bothrops jararacussu* metalloprotease. Additionally, partial inhibition of PLA_2_s activities from snake venoms was reported. The mechanism of action of EPema is still unknown, but it seems that proteolytic degradation as a potential mechanism can be excluded [36]. The aqueous extract prepared from *Schizolobium parahyba* (Sp) leaves, a native plant from Atlantic Forest (Brazil), inhibited 100% of lethality, hemorrhagic and indirect hemolytic activities of *Bothrops alternatus* and *Bothrops moojeni* snake venoms. Together with tannins, the extract also contains other compounds that can display specific inhibitory activity against snake venom toxins [37]. An aqueous extract from the leaves of *Casearia mariquitensis*, a plant found in Brazilian open pastures, displays components able to inhibit some hematological and systemic alterations induced by neuwiedase, a 22 kDa class P-I metalloproteinase from the venom of the pit viper *Bothrops neuwiedi pauloensis* [38,39].

Natural and artificial inhibitors of enzymatic, toxic and pharmacological effects induced by snake venom PLA_2_s are known, which act on PLA_2_s through different mechanisms, most of them still not completely understood. These include binding to specific domains, denaturation, modification of specific amino acid residues and others. Naturally occurring anti-toxic factors that neutralize PLA_2_ have been isolated from the blood of venomous snakes and studied [40,41,42]. Snake PLA_2_s inhibitors (PLIs) are large multimeric, serum proteins that form soluble complexes with PLA_2_ enzymes, thereby inhibiting their actions. The first PLIs were isolated from the serum of Habu snake, *Trimeresurus flavoviridis* (*Protobothrops flavoviridis;* EMBL Reptile Database) [43]. PLIs show specific affinities for various PLA_2_ enzymes and some have been shown to have anti-enzymatic, anti-myotoxic, anti-edema-inducing, anti-cytotoxic and anti-bacterial activities [44]. Fractionation of the serum of the venomous snake *Bothrops jararaca* resulted in the isolation of the anti-hemorrhagic factor BJ46a. BJ46a is a potent inhibitor of the SVMPs atrolysin-C (class P-I) and jararhagin (P-III) proteolytic activities and *B. jararaca* venom hemorrhagic activity. That was the first report of a complete cDNA sequence for an endogenous inhibitor of snake venom SVMPs. 

Recent studies on the functional basis of venom resistance show that blood serum from California ground squirrels can reduce the hemolytic activity of rattlesnake venom, as well as the activity of venom metalloproteases and other enzymatic toxins [45]. California ground squirrels were found to possess blood proteins that work as SVMP Inhibitors (SVMPIs). Those proteins are also present in the *Ig Supergene* family, but are not the same as SVMPIs from other species of mammals that are resistant to rattlesnake venom. 

### 3.2. Electric Current Treatment

In the late 1980s, a different approach was attempted to treat snake-bite injuries. Local application of high-voltage low-amperage direct electric current (dc) (so-called “electric shock for snakebites”) was reported by Guderian *et al.* [46] to be successful with 34 venomous snakebite injuries treated in the Amazon Jungle of Ecuador (where at the time poisonous snakebites were responsible for 4% of deaths). The treatment involved local application of a high voltage (20–25 kV) low amperage (<1 mA) dc for 1–2 s around the bite, from a portable system (“Stun-gun”) they had developed. The report claimed that 10 min after treatment, pain had gone and the sequelae of untreated bites (swelling, hemorragia, shock and renal failure, intense pain, edema, weakness, rapid pulse, bleeding disorders) did not develop [46]. A local effect or a direct effect on venom itself was proposed. A good experience with “Nova Spirit”, a Stun-gun, on a rattlesnake bite was reported [47]. Kroegel and Meyer zum Büshenfelde [48] speculated that electric shocks would influence the hydrogen bonds and metal ions of the venom enzymes, and reported a decrease in the histamine releasing activity of melittin after exposure of bee venom to high-voltage, but the biological basis for the treatment remains unknown. In field experiments with a so-called “Stun-gun”, it was reported that “all pain had gone” from the bitten limb [46]. 

Nowadays, a similar patent-pending device (ECOBITE, G. C. C., Switzerland) is commercialized in the USA and Europe for high-voltage electric low-amperage shock treatment of various poisonous bites (from insects, arthropods, and vertebrates), but its use is not recommended due to a lack of scientific basis [49]. Prevention is far preferable. Nevertheless, in many countries the serious problems posed by life-threatening venomous bites is a reality. 

Indeed, dc is largely employed in medical practice by a number of diverse empirical devices for treatment of inflammation and pain. For example, the Transcutaneous-Electrical-Stimulation (TENS) is an electronic device that produces electrical currents by connecting two or more electrodes to the skin [50]. TENS and other devices are empirical devices that seem to get good results, even though they still need a scientific validation. A recent report would offer a first scientific basis for the understanding of the mode of action of dc on enzymes [51]. In fact, the study of the interaction between dc and proteins is a poorly explored topic. When low (instead of high) voltages (0-10 V) were employed to expose reconstituted *Crotalus atrox* (Western Diamondback rattlesnake) venom in solution to dc (0-0.7 mA), it was found that after 1-5 seconds of exposure three of the most potent hemotoxic venom enzymes *i.e.*, Phosphatidate 2-acylhydrolase (phospholipase A_2_, PLA_2, _EC 3.1.1.4), phosphodiesterases (oligonucleate 5’-nucleotido hydrolase, exonuclease I, PDE, EC 3.1.4.1), and SVMPs, were irreversibly inactivated (>80%) [52]. As for what metalloproteinase inhibition is concerned, it was hypothesized that inactivation of *C. atrox* venom metalloproteases by dc application is due to displacement or removal the Zn^2+ ^ion from the enzyme active site, causing enzyme inactivation [52]. Chelation of the zinc atom by other means was reported to abolish both proteolytic and hemorrhagic effects of the venom [30].This would be consistent with the report from Kroegel and Meyer zum Büshenfelde [48], who supposed that electric current may influence metal ion moieties of some venom enzymes. Also, in field treatments with “stun gun” Guderian reported that ”the hemorrhagic sequelae of untreated poisonous bites were ruled out” [46].

Metal chelation can be accomplished in many ways: by treatment at low pH, or with chelating agents. For example, the lethal toxicity, of an hemorrhagic fibrinogenase toxin f (HT-f) isolated from *Crotalus atrox* venom containing 1 mol of zinc per mol of protein, was lost upon removal of Zn^2+ ^from the apo-HT-f, by treatment at low pH values or with chelating agents [31]. Acurhagin, a metalloproteinase from *Agkistrodon acutus* venom, was completely inactivated by treatment with metal chelators [53]. Citrate (100 mM) was found to inhibit *Crotalus adamanteus* venom metalloproteases and phosphodiesterases also *in vivo* [54]. PLA_2_ and metalloproteases with disintegrin domains are among the major venomous enzymatic components of hemotoxic *Viperidae* snake venoms [55].

For what the inhibition of *Crotalux atrox* PLA_2_ by dc exposure is concerned, the parameter CH-PO (CHarged minus POlar amino acids, *i.e.*, the percentage of charged residues subtracted from the percentage of polar residues of a protein [56]) seems to be related to the electro-inactivation of this protein [51]. Interestingly, many enzymes from venoms of various animals display a high CH-PO. venom PLA_2_ is one of these (CH-PO value: 12). A recent study reports that enzymes with CH-PO higher than 10.0 were irreversibly inactivated by dc; while those with CH-PO between +3 and –5 were not (CH-PO threshold at 10.0) [51]. Circular Dichroic spectroscopy showed a loss in ordered structure after PLA_2_ exposure to dc [49]. Therefore, it seems that a crucial role is played by dc in determining venom enzyme inactivation [51,52]. The topic of the treatment of venomous snake-bites by local application of low-voltage low-amperage electric current, may become clinically interesting for treatment of snake bites for both humans and animals (for example hunting dogs). Interestingly, ammodytoxin, a PLA_2_ from the venom of *Vipera ammodytes ammodytes* is a potent presynaptic neurotoxin [57]. It is tempting to speculate that the treatment with dc through the skin reported by Guderian *et al.* [44] had inactivated venom PLA_2_ [21].

Many thermostable proteins display a large difference between charged (Asp, Glu, Lys, Arg) *versus* polar (Asn, Gln, Ser, Thr) amino acids [56], and therefore a high CH-PO value. A network of ion bonds at the protein surface stabilizes many thermostable proteins, while the number of polar residues (tending to introduce the aqueous solvent into the core of the protein) is low [58]. Anyway, other factors contribute toward thermal stability of (*i.e.*, increase in secondary structure, in hydrophobic interactions, disulfide bonds and oligomerization) [59]. 

The advantages and disadvantages of the different ways to inhibit the hemorrhagic venoms are reviewed in Table 2.

More rigorous physical-chemical studies are needed for the interpretation of the interaction between dc and proteins at the molecular level. In turn, the research on the possible applications of compounds form plants of animals, capable of inhibit macromolecular components of snake venoms may supply helpful alternative or supplemental treatments to serum therapy. Undoubtedly, the perspective of the availability of affordable, inoffensive means to inactivate snake venoms *in vivo* is quite attractive.

**Table 2:** Advantages and disadvantages of different approaches to inhibit hemorrhagic venoms.

Approach for inhibition of hemorrhagic venom	Advantages	Disadvantages
Horse or sheep-derived polyclonal antivenins	Large-scale use	Adverse reaction or anaphylactic shock
	High efficacy	Economic and ethic problems
Sheep-derived antibody (CroFab)	Utilized	Economic and ethic problems
	High efficacy	
	Less allergenic	
Polyclonal antibodies purified from chicken egg yolks of immunized hens (*developed for Bothrops* sp. *venoms*)	Promising for the yield of antibody	Economic problems
	Probably less allergenic	Not utilized
		(protocols to be developed and efficacy tested)
Natural inhibitors	Probably less allergenic	Economic problems
		Not utilized (protocols to be developed)
Electric current treatment	Probably not allergenic	Not utilized nor recommended
	Applicable by a portable system	(lack of scientific basis)
